# Complete Genome Sequence of Lychnis Mottle Virus Isolated in Japan

**DOI:** 10.1128/genomeA.00535-18

**Published:** 2018-06-21

**Authors:** Yuji Fujimoto, Takamichi Nijo, Naoi Hosoe, Kiyoto Watanabe, Kensaku Maejima, Yasuyuki Yamaji, Shigetou Namba

**Affiliations:** a Department of Agricultural and Environmental Biology, Graduate School of Agricultural and Life Sciences, The University of Tokyo, Tokyo, Japan

## Abstract

We report here the first complete genome sequence of a Japanese isolate of lychnis mottle virus (LycMoV-J). The genome segments of LycMoV-J have a unique structure in their 3′ untranslated regions, and the encoded proteins have the same structure as that of an isolate reported from South Korea.

## GENOME ANNOUNCEMENT

Lychnis mottle virus (LycMoV) is a tentative member of the family Secoviridae, reported in Lychnis cognata in South Korea (1). The reported isolate has a bipartite genome consisting of two positive-strand RNAs, RNA1 (7,428 nucleotides [nt]) and RNA2 (3,734 nt), and each RNA molecule encodes a polyprotein (1). In Japan, although the partial sequence of LycMoV was detected in Rehmannia glutinosa (2), the complete genome sequence of LycMoV has not yet been determined.

In 2017, we collected Vincetoxicum acuminatum leaves showing mottle symptoms in Japan. Total RNA was extracted from the leaves using Sepasol-RNA I Super G solution (Nacalai Tesque, Japan) and treated with DNase I (Roche, Switzerland). A paired-end sequencing cDNA library was constructed from the extracted RNA using a TruSeq RNA sample prep kit version 2 (Illumina, USA) and sequenced on the MiSeq platform (Illumina) using a MiSeq reagent kit version 2 (500 cycles). The reads were *de novo* assembled using Trinity software version 2.4.0 (3). The assembled contigs were subjected to a BLASTn search against the GenBank database (4), and contigs showing sequence identity with the Korean LycMoV isolate (GenBank accession no. KR0110332 and KR011033) were obtained. To determine the complete genome sequence, undetermined regions were amplified by reverse transcription (RT)-PCR. The 5′ and 3′ terminal fragments were amplified using the 5′ RACE system for rapid amplification of cDNA ends kit version 2.0 (Invitrogen, USA) and RT-PCR with LycMoV-specific and oligo(dT) primers, respectively. The amplified fragments were cloned into a pCR-Blunt II-TOPO vector (Invitrogen), and eight clones were sequenced to determine the complete sequence.

Unlike the Korean LycMoV isolate, the 3′ untranslated region of each RNA in the Japanese isolate (LycMoV-J) contained an internal poly(U) region (nt 7170 to 7184 in RNA1 and nt 3642 to 3659 in RNA2), which was interposed between duplicated sequences (ca. 200 nt). The two strands of the complete genome, RNA1 and RNA2, were 7,478 and 3,953 nt long, respectively, excluding the poly(A) tails at their 3′ ends but including the 15- and 18-nt long poly(U) regions. RNA1 encoded a single polyprotein (nt 227 to 6844) predicted to be cleaved into a protease cofactor (Pro-C; nt 227 to 2317), a helicase (HEL; nt 2318 to 3961), a viral genome-linked protein (VPg; nt 3962 to 4045), a protease (Pro; nt 4046 to 4816), and an RNA-dependent RNA polymerase (RdRp; nt 4817 to 6844). RNA2 encoded a single polyprotein (nt 332 to 3316) predicted to be cleaved into a movement protein (MP; nt 332 to 1429), a large coat protein (LCP; nt 1430 to 2608), and a small coat protein (SCP; nt 2609 to 3316). The Pro-C of LycMoV-J contained 55 amino acid deletions in comparison to the Korean LycMoV isolate. The amino acid sequence identities of the LCP-SCP and Pro-Pol regions of LycMoV-J with those of the South Korean isolate were 93% and 94%, respectively. Therefore, based on the current classification criteria of the family Secoviridae (5), LycMoV-J was proven to be an isolate of LycMoV.

### Accession number(s).

The genome sequence of LycMoV-J has been deposited in DDBJ/GenBank under accession no. LC382242 and LC382243.
